# Ethnobotanical knowledge of Apiaceae family in Iran: A review

**Published:** 2016

**Authors:** Mohammad Sadegh Amiri, Mohammad Reza Joharchi

**Affiliations:** 1 *Department of Biology, Payame Noor University, Tehran, Iran*; 2 *Department of Botany, Research Center for Plant Sciences, Ferdowsi University of Mashhad, Mashhad, Iran*

**Keywords:** *Apiaceae*, *Ethnobotany*, *Medicinal Plants*, *Non- Medicinal Plants*, *Iran*

## Abstract

**Objective:**

Apiaceae (Umbelliferae) family is one of the biggest plant families on the earth. Iran has a huge diversity of Apiaceae members. This family possesses a range of compounds that have many biological activities. The members of this family are well known as vegetables, culinary and medicinal plants. Here, we present a review of ethnobotanical uses of Apiaceae plants by the Iranian people in order to provide a comprehensive documentation for future investigations.

**Materials and Methods:**

We checked scientific studies published in books and journals in various electronic databases (Medline, PubMed, Science Direct, Scopus and Google Scholar websites) from 1937 to 2015 and reviewed a total of 52 publications that provided information about different applications of these plant species in human and livestock.

**Results:**

As a result of this review, several ethnobotanical usages of 70 taxa, 17 of which were endemic, have been determined. These plants were used for medicinal and non-medicinal purposes. The most commonly used parts were fruits, leaves, aerial parts and gums. The most common methods of preparation were decoction, infusion and poultice.

**Conclusion:**

To our knowledge, this paper represents a comprehensive literature search of ethnobotanical uses of Apiaceae reported from Iran. This study highlights the rich traditional knowledge of this family that has remained in Iran. However, most of this knowledge survive only as memories from the past in the minds of the elderly, and will probably vanish in a few decades. Thus, we compiled these scattered data together in a single document for the next scientific works with ethnobotanical interests.

## Introduction

The Apiaceae (previously known as the Umbel Family: Umbelliferae) is one of the largest plant families in the world. This family comprises approximately 450 genera and 3700 species worldwide (Pimenov and Leonov, 1993 ▶). The members of this family are well known as vegetables, culinary and medicinal plants such as *Anethum graveolens *(dill)*, Anthriscus cerefolium *(chervil)*, Angelica spp.* (angelica)*, Apium gravolence *(celery)*, Carum carvi *(caraway)*, Coriandrum sativum *(coriander)*, Cuminum cyminum *(cumin)*, Foeniculum vulgare *(fennel)*, Ferula gummosa *(galbanum),and *Pimpinella anisum *(anise), *etc.* Plants of this family usually possess a characteristic pungent or aromatic smell which is owing to the presence of essential oil or oleoresin (Singh and Jain, 2007 ▶). Members of Apiaceae possess various compounds with many biological activities. Some of the main properties are ability to induce apoptosis, antibacterial, hepatoprotective, vaso-relaxant, cyclooxygenase inhibitory and antitumor activities (Pae et al., 2002 ▶). For the family Apiaceae, Iran is a major center of diversification. In Iran, the Apiaceae family is represented by 121 genera and 360 species. Iran with unique climatic conditions has a large variety of plants, especially some unique endemic plants. From the endemism points of view, Apiaceae is an important family in the flora of Iran with 122 endemic taxa (Mozaffarian, 2007 ▶; Emami and Aghazari, 2011 ▶). Iran has a very honorable history in folk medicine, which dates back to the time of Babylonian-Assyrian civilization. One of the most significant ancient heritages is knowledge of people who tried over the millennia to discover useful plants for health improvement and each generation added their own experience to this tradition (Naghibi et al., 2005 ▶). Iran has a long history of medical practice and knowledge of plant remedies. The documentation of traditions of plant use in Iran was begun many years ago (Hopper and Field, 1937 ▶). Recently, several local ethnobotanical studies focusing on different parts of Iran have been published (Amin, 1992 ▶; Zargari, 1996 ▶; Ghorbani, 2005 ▶; Ahvazi et al., 2012 ▶; Amiri et al., 2012 ▶; Emami et al., 2012 ▶; Mosaddegh et al., 2012 ▶;Rajaei et al., 2012 ▶; Amiri and Joharchi, 2013 ▶; Safa et al., 2013 ▶; Dolatkhahi and Nabipour, 2014 ▶; Sadeghi et al., 2014 ▶; Sharififar et al., 2014 ▶; Tahvilian et al., 2014 ▶; khodayari et al., 2015 ▶). However, there are no distinct references on the ethnobotanical applications of this family in Iran and most of the publications and documents are scattered. Thus, we compiled these scattered data together in a single document for the next scientific works with ethnobotanical interests. In addition, we reported information on conservation and endemism status of some of these taxa in Iran.

## Methods

We checked scientific studies in various electronic databases (Medline, Pubmed, Science Direct, Scopus, and Google Scholar websites) from 1937 to 2015. After a comprehensive search on the ethnobotanical aspects of Apiaceae family in Iran, we reviewed a total of 52 publications that provided information about different applications of these plant species in human and livestock. In this article, scientific and author names of plant species were checked for latest changes according to “The plant list” (http://www.theplantlist.org).

## Results

**Figure 1:**
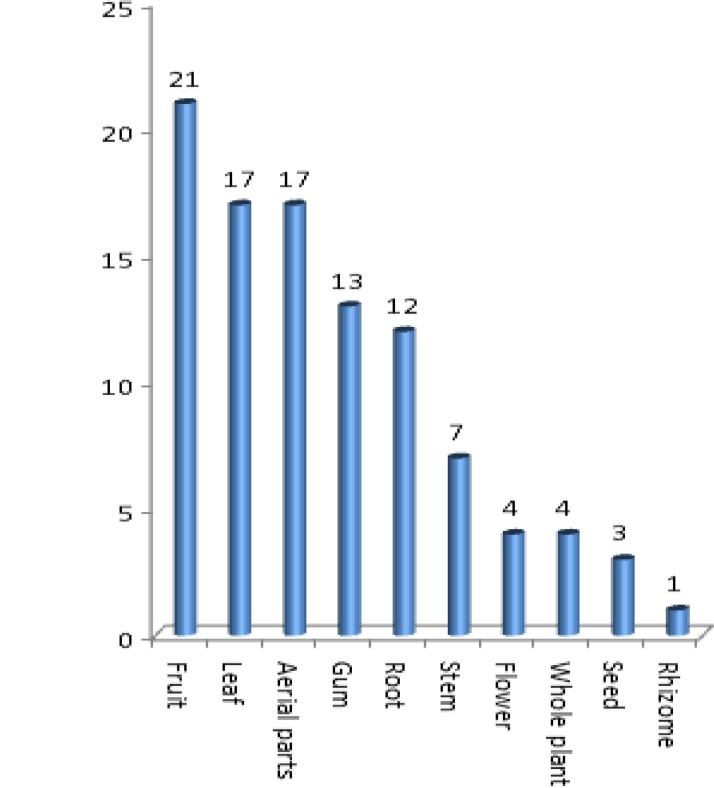
Proportional presentation of plant parts used

**Figure 2:**
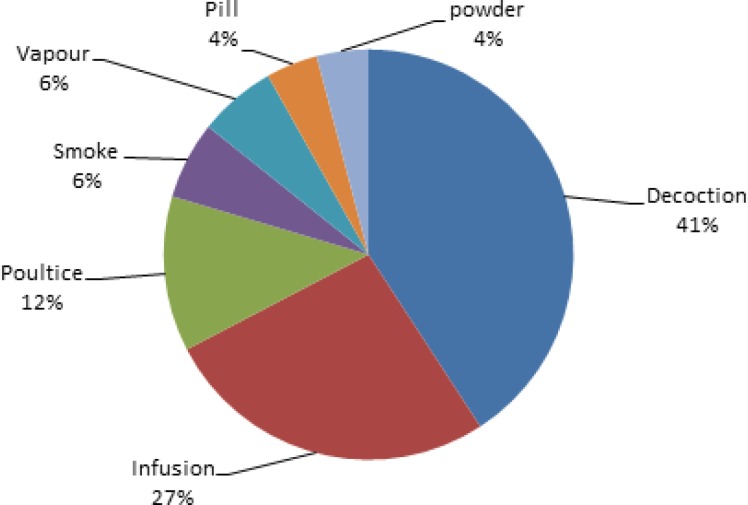
Mode of preparations and their percentages

In this review, ethnobotanical usages of 70 species, 17 of which were endemic, have been determined. Table 1 illustrates the results of this study. The plants used for various purposes in different parts of Iran were arranged in alphabetical order of their botanical names, with the relevant data. The information includes vernacular names, the part(s) used, the method of preparation, and medicinal and non-medicinal aspects along with literature sources. The species marked with an asterisk (*) were endemic species belonging to this family in Iran. The mostly used parts of the plants were fruits (21 species) followed by leaves (17 species), aerial parts (17 species), gum (13 species), root (12 species), stem (7 species), flowers (4 species), whole plant (4 species), seed (3 species) and rhizome (1 species) (Figure 1). The most common methods of preparation were decoction (20 species), followed by infusion (13 species), poultice (6 species), smoke (3 species), vapor (3 species), pill (2 species) and powder (2 species) (Figure 2).

**The importance of ethnobotanical aspects**

Ethnobotanical investigations generally result in the documentation of a rather limited set of well-documented beneficial plants, mostly medicinal, but also those known to be poisonous or used in nourishment (Ghorbani et al., 2006 ▶). In this paper, the members of Apiaceae family were used for various purposes, however we categorized their uses into three main groups including: 1) medicinal plants used in human; 2) medicinal plants used in livestock and 3) non-medicinal aspects.

**Medicinal plants used for humans**

From a total of 70 species belonging to this family, 66 species were reported to be used for medicinal purposes in human. Among them, the most frequently quoted species in this category were: *Bunium persicum* (Boiss.) B.Fedtsch., *Cuminum cyminum* L., *Dorema aucheri *Boiss., *Dorema ammoniacum *D.Don, *Ducrosia anethifolia* (DC.) Boiss., *Ferula assa-foetida* L., *Ferula gummosa *Boiss., *Ferulago angulata *(Schltdl.) Boiss., *Oliveria decumbens *Vent., *Prangos ferulacea* (L.) Lindl. and *Smyrnium cordifolium *Boiss. Most reported medicinal uses were for treatment of gastro-intestinal, respiratory system, urinary systemmetabolic system, gynaecological and skin disorders, and also they were used as antiseptic, anthelmintic, calmative, antipyretic, galactogogue and appetizer agents.

**Medicinal plants used for livestock**

Ten species have been recorded to have medicinal uses in veterinary. Among them, the most commonly used plants were: *Carum carvi *L. fruits, which were consumed for ectoparasites, digestive troubles, skin diseases, fever and mouth infection in livestock. The fruits of *Cuminum cyminum *L. were used to increase libido in female camels and as an anti-bloat agent in cattle and sheeps. Gum and root of *Dorema ammoniacum *D.Don were used to treat infectious wound infection and abscess in sheep and goat. Gum and root of *Dorema aucheri *Boiss. Were used for treatment of infectious wounds and infection in sheep. Aerial parts of *Oliveria decumbens *Vent. were used for treatment of diarrhea. Aerial parts and root of *Smyrnium cordifolium *Boiss. were used for treatment of urinary retention.

**Plants with non-medicinal uses**

From the 70 taxa recorded in this article, 30 species had both medicinal and non-medicinal applications. Apart from these, four species including *Astrodaucus orientalis *(L.) Drude, *Chaerophyllum macropodum *Boiss., *Froriepia subpinnata* (Ledeb.) Baill. and *Physospermum cornubiense *(L.) DC. had no medicinal effect and were only used for other purposes. In totally, thirty-four species have been reported for miscellaneous uses including edible, making pickles, as natural dyes and as flavors in salad, soup, *etc*. As stated in Table 1, the most cited species for edible uses were *Anethum graveolens* L., *Coriandrum sativum* L., *Cuminum cyminum *L., *Ferulago angulata *(Schltdl.) Boiss., *Foeniculum vulgare* Mill., *Heracleum persicum *Desf. ex Fisch., *Petroselinum crispum *(Mill.) Fuss, *Pimpinella anisum* L., *Prangos ferulacea *(L.)Lindl. and *Smyrnium cordifolium *Boiss. Many of these taxa were used all over the country. However, some other species, such as *Dorema aucheri *Boiss., *Kelussia odoratissima *Mozaff. and *Oliveria decumbens *Vent. were only used in a small area. Most of these taxa were used as wild vegetables. These species with much narrower distribution were exclusively used in Iran, and therefore could be considered as ‘typical Iranian wild edibles’. It is sometimes hard to know whether a particular sample was wild or cultivated. For example, some species, such as *Kelussia odoratissima *Mozaff. were only native to certain parts of Iran, although they were cultivated in some other regions of the country. Kelus or karafs-e-Bakhtyari (*Kelussia odoratissima *Mozaff.) was widely used as a wild vegetable and flavoring. It could be found in local markets and beside the roads by local people. Some species such as *Echinophora platyloba *DC., *Kelussia odoratissima *Mozaff. and *Levisticum officinale *W. D. Koch were used as a flavor in yoghurt. *Chaerophyllum macropodum *Boiss. was cooked and eaten with rice. *Ferula angulata *Schltdl. was added as a flavor to animal oil. The young leaves and branches of *Dorema aucheri *Boiss. were used for making a locally famous pickle called “Bilhar Pickle” and used as vegetable in a local soup. Non-edible uses have also been recorded. For instance, aerial parts of *Prangos ferulacea *(L.) Lindl. were used as a dye (yellow color). 

**Comments on some most cited species**

Our results indicated that medicinal species such as *Bunium persicum* (Boiss.) B.Fedtsch., *Dorema ammoniacum *D.Don, *Ducrosia anethifolia* (DC.) Boiss., *Ferula assa-foetida* L., *Ferula gummosa *Boiss., *Oliveria decumbens *Vent., *Prangos ferulacea* (L.) Lindl. and *Smyrnium cordifolium *Boiss. were mentioned by many studies. Among Iranian people, the use of Zireh(Persian name) is very popular. According to Table 1, five kinds of Zireh including Zireh-e-Siah (*Bunium persicum* (Boiss.) B.Fedtsch.), Zireh-e-Shami (*Carum carvi *L.), Zireh-e-Sabz (*Cuminum cyminum* L.), Zireh-e-Sefid (*Cuminum setifolium *(Boiss.) Kos.-Pol.) and Zireh-e-vahshi (*Lagoecia cuminoides *L.)were used in Iran. The most remarkable one that has the highest number of reports was *Bunium persicum* (Boiss.) B.Fedtsch., which is also known as Zireh-e-koohi in Iran. In Iranian folk medicine, this species was applied as as a galactogogue, carminative, calmative, appetizer, decongestant agent and to treat indigestion, children earache, newly delivered ladies recovery, cold-natured conditions and weaknesses. In addition, it was used as a flavor. The genus *Ferula *comprises about 170 species distributed from central Asia to northern Africa. It represented by 30 taxa, 20 of which are endemic to Iran. The popular Persian name for most of these species is “Koma” (Pimenov and Leonov, 1993 ▶; Emami and Aghazari, 2011 ▶; **Mozaffarian, 2007**). Most species of this genus have been used in traditional medicine. The most striking of them, with the highest number of citations were* Ferula assa-foetida* L. and *Ferula gummosa *Boiss. The most popular Persian names for *Ferula assa-foetida* L. were Anghuzeh, Heltit and Gane-bu. It was claimed to be highly effective on stomachache, cough, epilepsy, tremor and epilepsy and is used as an anthelmintic and antihemorrhoid agent and also in the treatment of gastritis. *Ferula gummosa *Boiss., commonly known as Barijeh or Ghasni, was used for liver cysts and dyspepsia, and as an anthelmintic, anticatarrhal, antiallergic, appetizer and emmenagogue agent. The genus *Dorema* is represented by 7 species in Iran, 2 of which are endemic. The most famous of them, with the highest number of citations were *Dorema ammoniacum *D.Don and *Dorema aucheri *Boiss. The most popular persian names for *Dorema ammoniacum *D.Don were Kandal, Vasha and Ushegh. It was traditionally used for the treatment of different diseases, such as cystitis, digestive, colic, furuncles, and asthma and as an anthelmintic, emmenagogue and anticovulsion agent. In Iranian traditional medicine, *Dorema aucheri *Boiss. was used against asthma, bronchitis, parasites of digestive system, constipation and burns. The genus *Oliveria *is represented only by a single species, namely *Oliveria decumbens *Vent., in Iran. It was traditionally used for the treatment of different diseases such as indigestion, diarrhea, abdominal pain, feverish conditions, stomach pain and cold and to relieve thirst in children. 

**Credibility of plant species used in ethnobotany**

Due to the interdisciplinary nature of ethnobotany, few individuals can be expected to be experts in all components of the cross-disciplinary research that ethnobotany represents in botany, pharmacology, medicine, chemistry, anthropology and linguistics. Therefore, it needs a close collaboration of multidisciplinary teams of researchers who are experts in botany, pharmacology, medicine and anthropology (Alexiades and Sheldon, 1996 ▶). Unfortunately, in Iran, botanists are not really involved in this field of inquiry, even though botany is one of the basic fields involved in interdisciplinary field of ethnobotany. Most of the studies in Iran have been done by pharmacognosists and anthropologists. Improvement of ethnobotany and ethnopharmacology in Iran needs more involvement of botanist in these fields (Ghorbani et al., 2006). Sometimes, the studies of ethnobotany, can comprise a few incorrect identifications. Botanists attempt to record a much lower number of erroneous ethnobotanical taxa. Reports on plants which do not exist in Iran may be a result of plant misidentification. For example, in the literature review of this family in Iran, we found that *Eryngium campestre* L. has been recorded for ethnobotanical uses (Mirdeilami et al., 2011 ▶). However, according to Flora Iranica, this species does not exist in Iran (Mozaffarian, 2007 ▶). Therefore, we have removed this plant from the list (Table 1). In some cases, identical names are given to different species, or various names to the same species. This is particularly important for taxa that are marketed. Owing to some morphological similarities of the plant parts and their improper identification by the consumers and herbal plant sellers and lack of a standard identification system, the crude medicinal plants and their parts are often adulterated or substituted in commerce which may result in the loss of their efficacy. For instance, *Zosima absinthifolia *Link adulterated or substituted instead of *Levisticum officinale *W.D.J.Koch in some commercial samples. Identification and recognition of medicinal plants are very important because the adulterants, although belonging to the same genus, do not possess the medicinal properties of the drug. For example, *Bunium cylindricum *is being mixed with real Zireh-e-siah (*Bunium persicum*) and is sold in the market but with less quality and efficacy. Correct identification of plant species is the foundation of safe use of herbal medicines and products. Therefore, in order to ensure safety, therapeutic potency and efficacy of lucrative and medicinal plants, correct identification, authentication, and elimination of adulteration are essential and the taxa should only be authenticated by a panel of experts including taxonomists (Joharchi and Amiri, 2012 ▶). 

**Comments on conservation status of some notable species**

Some of species have a narrow distribution and collection from wild populations will threaten these taxa. Furthermore, various parts of plants should only be collected in such a manner that ensures their continued presence, both in specific collection locations and across the landscape (Meeker et al., 1993 ▶). Harvesting from wild populations and destructive collecting methods, such as removal of subterranean and aerial parts which are essential to the survival of the plants, could be serious threats and often lead to vanish this species. Various species of Apiaceae family are monocarpic, so that only once produce flowers during the life cycle and only reproduce through seeds such as* Ferula spp*. Excessive harvest of roots and flowers of these species are dangerous, and must be avoided, especially in the case of endemic and endangered species. Of the 70 plant species included in this review, 17 taxa were listed as endemic**. **Some of these species such as *Dorema aucheri *Boiss., *Echinophora cinerea* (Boiss.) Hedge &Lamond, *Ferula hezarlalehzarica *Ajani and *Kelussia odoratissima *Mozaff. are narrow-range endemics and occur only in a few specialized niches. *Heracleum gorganicum *Rech.f. is an endemic species of Iran which is widely and heavily harvested from the wild and this could be a big threat for these species (Ghorbani, 2005 ▶). *Dorema aucheri *Boiss. is considered as an endemic species which is heavily collected. Excessive collection of these plants has caused a notable decrease in populations of the plant in the area. Many individuals of young plants are harvested to be sold (Mosaddegh et al., 2012 ▶). *Kelussia odoratissima *Mozaff. is another endemic species with a narrow distribution range in Iran which is subjected to heavy use by inhabitants of the region. The local people are using the whole plant for different purposes. Excessive collection of it has led to the decrease of the plant in the area. Some rare species such as *Levisticum officinale *and *Dorema ammoniacum *have been threatened as herbalists and traders hire the local people for collecting these species due to the economic purposes. In addition, local people sometimes sell these medicinal plants in the local market for making money (Rajaei et al., 2012 ▶). Many of these plants are potentially endangered and vulnerable taxa. Collecting of plants from the wild led to the impoverishment of various plant communities in many areas, especially for plants that their roots or flowers are used, and that harvesting should be controlled. So, sustainable harvesting and domestication of these plants is a need for conservation which would guarantee these renewable resources for the future. Special consideration should be given to promising plants in the area and protect them from extinction by excessive utilization.

**Table 1:** Importance of ethnobotanical applications of Apiaceae family in Iran

Reference cited	Non-medicinal uses	Medicinal uses (Livestock)	Medicinal uses (Human)	Preparation	Part used	Vernacular name	Scientific name	NO
Safa et al., 2013; Dolatkhahi et al., 2012;	-	-	Flatulency, Diuretic, Tonic, digestant, dyspepsia	**-**	Fruit	Khelale-dandan	*Ammi majus* L.	1
khodayari et al., 2015.	-	-	Tonic, treatment ofgingivitis, Digestive disorders, Carminative, appetizer	**-**	Aerial parts	Khelale-dandan	*Ammi visnaga* (L.) Lam.	2
Amiri and Joharchi, 2013; Ghorbani, 2005; Sharififar et al., 2010; Dolatkhahi et al., 2012; Sadeghi and Mahmood, 2014; Azizi and Keshavarzi, 2015; Dolatkhahi and Nabipour, 2014;	Culinary	-	Abortion, Anti-Dysmenorrhea, Galactogogue, Antihyperlipidemia, Carminative, Treatment of Diabetes, Digestive disorders, Infertility treatmentmen	Infusion	Fruit- Shoot	Shevid, Toragh	*Anethum graveolens* L.	3
khodayari et al.; 2015.	-	-	Treatment stomachache,Antihyperlipidemia,Stomachtonic	**-**	Leaf; root	Jenjil	*Anthriscus sylvestris* (L.) Hoffm	4
Hopper and Field, 1937; Amin, 1992; Amiri and Joharchi, 2013; Ghorbani, 2005; Mardaninejad et al., 2013;	Culinary	-	Carminative, Tonic, Emmenagogue, Diuretic, Liver disorders, asthma, Loss of appetite, Rheumatic, Lumbago	Decoction	Fruit, Leaf; Stem	Karafs	*Apium graveolens* L.	5
Nazemiyeh et al., 2009.	used as a salad, vegetable and a food additive	-	**-**	**-**	Whole plant	Havij-e-kohi	*Astrodaucus orientalis *(L.) Drude	6
Amiri and Joharchi, 2013; Safa et al., 2013; khodayari et al., 2015; Sharififar et al., 2010; Amiri et al., 2012.	Flavoring	-	Obesity, Galactogogue, Flavoring, Carminative, Calmative, Appetizer, Indigestion,decongestant,children earache, newly delivered ladies recovery,cold-natured treatment, strengthening weaknesses	Decoction, powder	Fruit	Zireh-e-Siah	*Bunium persicum* (Boiss.) B.Fedtsch.	7
Rajaei et al., 2012.	-	-	Fever, dermal wound, Joint pain and inflammations	Decoction, poultice	Leaf; seed	-	*Bupleurum falcatum *L.	8
Mardaninejad et al., 2013; Sadeghi and Mahmood, 2014; Ghorbani et al., 2014.	Flavoring	Ectoparasites, Digestive and Gastric troubles, Skin diseases, Fever, Dehydration, Mouth infection	Obesity, Facilitate digestion, Sour stomach, Blood pressure, Diarrhea	Infusion	Fruit	Zireh-e-Shami	*Carum carvi *L.	9
Mosaddegh et al., 2012.	Eaten with rice	-	**-**	**-**	Young stem	Garkava, Chelghaba	*Chaerophyllum macropodum *Boiss.	10
Hopper and Field, 1937; Amin, 1992; Amiri et al., 2014; Ghorbani, 2005; Tahvilian et al., 2014; Mardaninejad et al., 2013; Azizi and Keshavarzi, 2015.	Flavoring,culinary	-	Relieve headache, relieve toothache jaundice, Acne, Treat of Flatulence, Appetizer, Aphrodisiac, Antiseptic,Gasteralgia, sore throat, Aromatic, Painkiller,Diabetescontrol,Gout control	Decoction	Fruit	Geshniz,Geshnij	*Coriandrum sativum* L.	11
Amiri and Joharchi, 2013.	-	-	Cholagogue, Depilator, Treat of Dermal Allergies	**-**	Root	Shokaran	*Conium maculatum* L.	12
Hopper and Field, 1937; Amin, 1992; Amiri and Joharchi, 2013; Sharififar et al., 2010; Koohpayeh et al., 2011;	Flavoring,culinary	Increase libido female camel, Anti-bloat in cattle and sheep	Relieve pain after child-bitth, Carminative, Treat of Colic, Galactogogue, Obesity, Digestive, Favoring, Antiasetic	Infusion	Fruit	Zireh-e-Sabz	*Cuminum cyminum* L.	13
Safarnejad et al., 2011.	Spice	-	Carminative	**-**	Fruit	Zireh-e-Sefid	*Cuminum setifolium *(Boiss.) Kos.-Pol.	14
Amiri and Joharchi, 2013; Sharififar et al., 2010; khodayari et al., 2015; Ghorbani et al., 2014.	Edible, culinary	Endoparasites	Diuretic, Emmenagogue,Disposalofworms,constipation, appetizerand Diuretic	**-**	Fruit, root	Havij	*Daucus carota* L.	15
Hopper and Field, 1937; Amin, 1992; Amiri and Joharchi, 2013; Rajaei et al., 2012; khodayari et al., 2015; Sadeghi and Mahmood, 2014; Koohpayeh et al., 2011.	-	Infectious wound healing and infection, Abscess in the sheep and goat	Indolent tumors, cystitis, Digestive, treat of colic, Treat of Furuncles, laxative, Expectoran, Asthma, Anthelmintic, Emmenagogue, Anticovulsion	Infusion, poultice	Gum, root	Kandal,Vasha, Ushegh	* *Dorema ammoniacum *D.Don	16
Rajaei et al., 2012; Mosaddegh et al.; 2012; Tahvilian et al., 2014; Koohpayeh et al., 2011; Mozaffarian, 2013.	Edible, use as vegetable, young stems are pickled	Infectious wound healing and infection in sheep	Asthma, Expectorant, Bronchitis, Making Gum,Parasites of digestive system, constipation, Burn healing	Fresh paste	Gum, young aerial part, root	Kal, Bilhar	* *Dorema aucheri *Boiss.	17
Sadeghi and Mahmood, 2014; Sadeghi et al., 2014.	-	-	Abortion, aphrodisiac, Scorch	Decoction, cataplasm	Gum	Oshtork	*Dorema aureum* Stocks	18
Delnavazi et al., 2015.	Used as a green vegetable	-	Diuretic, anti-diarrheal, treatment of bronchitis and catarrh	**-**	Leaves, gum-resin	**-**	*Dorema glabrum *Fisch. & C.A.Mey.	19
Rajaei et al., 2012; Sadeghi and Mahmood, 2014; Sharififar et al., 2014; Dolatkhahiand Nabipour, 2014.	-	-	Carminative, Irregularities of Menstruation, lactiferous	Decoction	Aerial parts, leaf, seed	Gicho,Goatk, Mashgak, Baghiz	*Ducrosia anethifolia *(DC.) Boiss*.*	20
Shafie-zadeh, 2002.	Spice	-	Stimulant and an invigorator of the stomach,diuretic,anti-cancer	-	Aerial parts	Phyaleh	* *Echinophora cinerea* (Boiss.) Hedge & Lamond	21
Tahvilian et al., 2014; Pirbaloutiet al., 2010; Abbasi et al., 2012.	Spice, flavoring with yogurt, culinary	-	Dissolves renal calculi, Anti aphthous (Mouth wash), antifungal	Decoction	Aerial parts	Khosharizeh	* *Echinophora platyloba *DC.	22
Mosaddegh et al., 2012; Abbasi et al., 2012; Sharififar et al., 2014.	-	-	Constipation, palliative, antifungal, arthritis pain reliever	Decoction	Aerial parts, root	Zole,kharzul, Chichagh	*Eryngium billardieri *Delile	23
Sharififar et al., 2014.	-	-	Painkiller	**-**	Aerial parts	Zole-e-Khorasani	*Eryngium bungei *Boiss.	24
Amiri and Joharchi, 2013; Tahvilian et al., 2014.	-	-	Treat of Vitiligo, Cut, Wound,Carminative, Febrifuge, Hemostatic	Decoction	Leaves,fruit	GhazYaghi,Paghaze	*Falcaria vulgaris *Bernh.	25
Emami et al., 2012; Safa et al., 2013; Mosaddegh et al., 2012; khodayari et al., 2015; Sharififar et al., 2010; Sadeghi and Mahmood, 2014; Sajjadi et al., 2011.	Culinary	-	Stomachache,Anthelmintic, Antihemorrhoid, Cough, Tremor,epilepsy, treatment of gastritis	Decoction	Gum	Anghuzeh, Heltit, Gane-bu	* *Ferula assa-foetida *L.	26
Zargari, 1996.	-	-	Anticonvulsant, tonic, anti-hysteric, decongestant, treatment of neurological disorders, stomach ache	**-**	Resin	**-**	*Ferula badrakema *Koso-Pol.	27
Bahmani et al., 2012; Pirbalouti et al., 2013.	-	Appetizer	Anti-septic	Smoking, Sodden	Stem, leaves, inflorescence	Kame,Anio	* *Ferula behboudiana *(Rech.f. & Esfand.) D.F.Chamb.	28
Zargari, 1996.	-	-	Anticonvulsant, tonic, anti-hysteric, decongestant, treatment of neurological disorders, stomach ache	**-**	Root	**-**	*Ferula diversivittata* Regel & Schmalh.	29
Amiri and Joharchi, 2013.	-	-	Anthelmintic, Treat of Colic,Emmenagogue	**-**	Gum	Anghuzeh	*Ferula foetida *(Bunge) Regel	30
Hopper and Field, 1937; Amiri and Joharchi, 2013; Ghorbani, 2005; Mosaddegh et al., 2012; khodayari et al., 2015; Amiri et al., 2012.	Powdered fruits, stem as pickle	-	Appetizer, treatment of wounds, liver cysts, Anthelmintic, Anticatarrhal, Antiallergic, Dyspepsia, Emmenagogue	Decoction, poultice	Fruit, gum, root	Barijeh,Angiyun,Ghasni	*Ferula gummosa *Boiss.	31
Pirbalouti et al., 2013.	-	-	Anti-septic	smoking	Stem, leaves, inflorescence	Komeh, Komieh	*Ferula haussknechtii *H.Wolff ex Rech.f.	32
Rajaei et al., 2012.	-	-	Stomachache, Carminative	Hydrodistilation	Stem, rhizome		* *Ferula hezarlalehzarica *Ajani	33
Amiri et al., 2012.	-	-	Indigestion and anthelmintic	**-**	Leaves		* *Ferula latisecta *Rech.f. & Aellen	34
Khodayari et al., 2015.	-	-	Anti-nausea, anti-stomach acid	**-**	Leaves, stem	Chevil	* *Ferula macrocolea *Boiss.	35
Ghorbani, 2005; Sharififar et al., 2014.	-	-	Cough, asthma, respiratory disorders, migraine, expectorant	Demulcent, vapor, pill	Seed, gum	Ejek-ghamaghi, kal	*Ferula oopoda *(Boiss. & Buhse) Boiss.	36
Abbasi et al., 2012; Ahmadi et al., 2009; Sajjadi et al., 2011.	-	-	Anticonvulsants, Tonic, constipation,Back pain treatment	Decoction	Shoot, fruit	Kama	*Ferula ovina *(Boiss.) Boiss.	37
Hopper and Field, 1937; Ahvazi et al., 2012; Sahranavard et al., 2014; Sharififar et al., 2014.	Steam cooked,Spicy, cooking, edible	-	Lumbago, rheumatism, gout, sinusitis, pododynia, backache,Treat epilepsy, laxative, antitussive	Poultice, vapor	Stems, roots, leaves, gum	Sakbinaj, Jarand	* *Ferula persica *Willd*.*	38
Ghorbani, 2005; Sharififar et al., 2010.	-	-	Asthma, cough, dermal wounds,stomach pain	Demulcent, vapor, pill	Gum	Ghamagh-mumi, Anghozeshirin	*Ferula szowitziana *DC*.*	39
Mosaddegh et al., 2012; Ahmadi et al., 2009; Pirbalouti et al., 2013;	As aromatic ingredient, as flavor in animal oil, spice and air fresher	Relieve flatulence	Anti-septic,renal pain	**-**	Leaves	Chavil, Chavir	*Ferulago angulata *(Schltdl.) Boiss.	40
Rajaei et al., 2012.	-	-	Dermal wounds	Poultice	Gum	Garchik	* *Ferulago carduchorum *Boiss. & Hausskn. ex Boiss.	41
Hopper and Field, 1937; Amin, 1992; Amiri and Joharchi, 2013; Ghorbani, 2005; Mosaddegh et al., 2012; khodayari et al., 2015; Sharififar et al., 2010; Sadeghi and Mahmood, 2014;	Edible, flavoring	-	Relieve toothache, Dysentery, Cold, Diuretic, kidney infections, Galactogogue, Digestive, Bronchitis, Appetizer, Antiacid, Flatulence,hypnotic	Decoction	Aerial parts, Fruit	Raajuneh, Razianeh	*Foeniculum vulgare* Mill.	42
Mozaffarian, 2013.	As a local vegetable, as a local spice, flavoring	-	-	-	Aerial parts	Zolang	*Froriepia subpinnata* (Ledeb.) Baill.	43
Ahvazi et al., 2012; Yazdanshenas et al., 2015; Sonboli et al., 2005.	Edible, cooking some foods,as a local vegetable and flavoring in soups and foods	-	Tonic, carminative and relief stomachache	Infusion	Leaves	Jafarikohi, Samoureh	*Grammosciadium platycarpum *Boiss. & Hausskn.	44
Mosaddegh et al., 2012.	-	-	Diabetes, hypertension	-	Aerial parts	Kelos-e kuhi	* *Haussknechtia elymaitica *Boiss*.*	45
Ghorbani, 2005.	Flavoring	-	Digestive disorders	**-**	Seed	Jengel-ghamaghi	* *Heracleum gorganicum *Rech.f.	46
Amiri and Joharchi, 2013; Ahvazi et al., 2012; khodayari et al., 2015.	Spice, flavoring	-	Treat of Hiccup, Appetizer, Flavoring, Carminative, Anthelmintic, Stomach Tonic,Tremor, migraine, headache caused by sinusitis	Infusion, decoction	Fruit, flowers	Golpar	*Heracleum persicum *Desf. ex Fisch.,C.A.Mey. & Avé-Lall.	47
Tahvilian et al., 2014.	-	-	Dissolves renal calculi, cornicide	Decoction	Leaf, root	Baraza	*Johrenia aromatic *Rech.f.	48
Pirbalouti et al., 2010; khodayari et al., 2015.	Edible as vegetable, Flavoring with yogurt	-	Indigestion, rheumatism, Gastric ulcer, anti-diabetes, pain, cough, Irritation, Sedative	**-**	Whole plant	Kelus, karafs-e-Bakhtyari	* *Kelussia odoratissima *Mozaff.	49
Safaet al., 2013; Mosaddegh et al., 2012; Dolatkhahi and Nabipour, 2014.	-	-	Bile stone repellent, Diarrhea	Infusion	Aerial parts	Alaf-e kaaji, Zireh-e-vahshi	*Lagoecia cuminoides *L.	50
Amiri and Joharchi, 2013; Rajaei et al., 2012.	Flavoring with yogurt, use as vegetable	-	Nerve Diseases, Heart Tonic, Indigestion,Blood sugar, Asthma, diuretic	Infusion	Fruit, leaf, root	Angedane-roomi, Karafse-kuhi	*Levisticum officinale *W.D.J.Koch	51
Mosaddegh et al., 2012; khodayari et al., 2015; Dolatkhahi et al., 2012; Bahmani et al., 2012; Dolatkhahi and Nabipour, 2014.	Culinary, use as vegetable	Diarrhea	Relieve thirst in children, indigestion, diarrhea, abdominal pain and feverish conditions, Stomach pain, cold therapy	Decoction	Aerial parts	Moshkurak, Tighnak, Den	* *Oliveria decumbens *Vent.	52
Pirbalouti et al., 2013.	-	-	Anti-septic	Smoking	Stem, leaves, inflorescence	Alafshir	*Opopanax hispidus *(Friv.) Griseb.	53
Amiri and Joharchi, 2013; Mardaninejad et al., 2013	Edible as vegetable, flavoring	-	Emmenagogue, Diuretic, Carminative, Kidney Disorders, Bladder disease, Gout, Blood pressure, Blood sugar, Varicocele	Infusion	Fruit	Jafari	*Petroselinum crispum *(Mill.) Fuss	54
Zarshenas et al., 2013.	-	-	Diuretic, Cough, Meningitis, Paralysis, Renal stone, Respiratory ulcers	**-**	Gum	Bokhurol ekrad	*Peucedanum officinale *L.	55
Alavi et al., 2005.	-	-	Treatment of cold	**-**	Fruit	Razianekoohi	*Peucedanum ruthenicum *M.Bieb.	56
Mirdeilami et al., 2011.	Edible	-	-	**-**	Stem	Ghaziaghi	*Physospermum cornubiense *(L.) DC.	57
Hopper and Field, 1937; Amin, 1992; Pirbalouti et al., 2013; Amiri and Joharchi, 2013; Sadeghi and Mahmood, 2014.	Culinary use	-	Treat of Flatulence, Anthelmintic,Treat of Colic, Antacid, Stomachache, Antidiarrhea	Infusion	Fruit	Vavehshing, Anison (Badianroomi)	*Pimpinella anisum* L.	58
Sharififar et al., 2014.	-	-	Treatment of flatulency	**-**	Aerial parts	Sakbinj	* *Prangos cheilanthifolia *Boiss.	59
Pirbalouti et al., 2013; khodayari et al., 2015; Azizi and Keshavarzi, 2015; Barani and Rahimpour, 2014; Ghorbani et al., 2014.	As a natural dye	Treatment of thick and louse (Ruminants)		Decoction	Aerial parts	Bale har, Ginoo, Marzah	*Prangos ferulacea *(L.) Lindl.	60
Sajjadi et al., 2011.	-	-	Tonic,Carminative	**-**	Fruit, flower	Jashir-e-sakhrehrooy	*Prangos uloptera* DC.	61
Sajjadi et al., 2011.	Flavoring	-	Disinfectants	**-**	Aerial parts	Shen jar	* *Psammogeton canescens *Vatke	62
Safa et al., 2013.	-	-	Back, leg and other part muscles pain	**-**	Leaves, stem	Sagdandan	*Pycnocycla aucherana *Decne. ex Boiss.	63
Mosaddegh et al., 2012.	-	-	Palpitation, blood coagulation, body pains	Decoction	Aerial parts	Suzanak	*Scandix pecten-veneris *L.	64
Sharififar et al., 2014.	-	-	Stomach tonic, Has a hot temper	**-**	Whole plant	Badian-e-koohi	*Scandix stellata *Banks& Sol.	65
Sahranavard et al., 2014;	-	-	Treat epilepsy	**-**	Whole parts	Sisalius	*Seseli tortuosum *L.	66
Mosaddegh et al., 2012; Ahvazi et al., 2012; Tahvilian et al., 2014; Pirbalouti et al., 2013; Bahmani et al., 2012; Ahmadi et al., 2009.	Roots and stems as a food to be consumed raw or cooked	Urinary retention	Urinary ducts and prostate problems, gynaecologicaldisease,Indigestion and stomachic,Bitter aromatic, hot effects, tonic,anti- helmintic,Antipyretic, anti-worm tooth	Infusion, Sodden	Aerial parts, seeds, root	Ovandol, Pinoume, Gonour	*Smyrnium cordifolium *Boiss.	67
Pirbalouti et al., 2010.	Spice and condiment	-	Anti-septic	**-**	Fruit	Goolpar, Kereson	*Tetrataenium lasiopetalum *(Boiss.) Manden.	68
Amiri and Joharchi, 2013; Sharififar et al., 2010.	Flavoring	-	Carminative, Anthelmintic, Antidiarrhea, Treat of Colic, Antacid, Galactogogue	Infusion	Fruit	Zenyan (Khordaneh), Kasrak	*Trachyspermum ammi *(L.) Sprague	69
Mosaddegh et al., 2012.	-	-	Urinary duct problems	Infusion	Aerial parts	Darehjouyi	*Turgenia latifolia *(L.) Hoffm.	70

(*) The species marked with an asterisk are endemic species belonging to Apiaceae family in Iran

## Conclusion

This paper clearly represents a deep-rooted ethnobotanical heritage of Apiaceae family in Iran. Traditional knowledge of Iranian peoples is based on oral tradition passed through several generations and most of this information survives only in the memory of the elderly people and is now in danger of vanishing. This review illustrates the necessity of ethnobotanical works in various regions of Iran to record all the folkloric knowledge practiced among indigenous people and attempts to compile these scattered data in order to help maintaining cultural traditions. The best and quickest way to species selection for pharmacological and phytochemical works is by reviewing the ethnobotanical literature. This highlights the significance of such investigations. Based on the data of this paper some taxa should be given priority for further phytochemical and pharmacological studies, including: *Dorema glabrum *Fisch. & C.A.Mey., *Echinophora cinerea *(Boiss.) Hedge & Lamond, *Johrenia aromatic *Rech.f., *Opopanax hispidus *(Friv.) Griseb. and *Pycnocycla aucherana *Decne. ex Boiss. Some species are good candidates for future research, specially in the case of endemic species. The flora of Iran is rich in endemic species of Apiaceae (122 taxa), many of which have been poorly investigated. These taxa are unique and potentially interesting as a basis for future research works. To our knowledge, there is no literature on some notable species that have been traditionally used in Iran such as *Azilia eryngioides* (Pau) Hedge & Lamond, *Ferula macrocolea *Boiss., *Haussknechtia elymaitica *Boiss., *Heracleum gorganicum *Rech.f., *Kalakia marginata* (Boiss.) Alava, *etc*. Identification of plants in each area provides a better understanding of restorable natural resources and their applications. Ethnobotanical efforts should continue, particularly in regions that have received less attention. It is strongly believed that detailed data as introduced in this paper on the ethnobotany of Apiaceae, provides detailed evidence for the use of these plants for different purposes*.* Regarding the rich background of traditional knowledge of these species, it seems there are still a large number of unaccomplished researches, which provides baseline data for subsequent pharmacological and phytochemical investigations. 

## References

[B1] Abbasi S, Afsharzadeh S, Mohajeri A (2012). Ethnobotanical study of medicinal plants in Natanz region (Kashan), Iran. J Herbal Drugs.

[B2] Ahvazi M, Khalighi-Sigaroodi F, Charkhchiyan MM, Mojab F, Mozaffarian VA, Zakeri H (2012). Introduction of medicinal plants species with the most traditional usage in Alamut region. Iranian J Pharm Res.

[B3] Alavi SHR, Yassa N, Fazeli MR (2005). Chemical constituents and antibacterial activity of essential oil of Peucedanum ruthenicum M. Bieb. fruits. Iranian J Pharm Sci.

[B4] Alexiades NM, Sheldon JW (1996). Selected guidelines for ethnobotanical research: a field manual.

[B5] Amin G (1992). Popular Medicinal Plants of Iran. Vol. 1, Research Deputy, Ministry of Health, Treatment and Medical Education, Tehran.

[B6] Amiri MS, Jabbarzadeh P, Akhondi M (2012). An ethnobotanical survey of medicinal plants used by indigenous people in Zangelanlo district, Northeast Iran. J Med Plants Res.

[B7] Amiri MS, Joharchi MR (2013). Ethnobotanical investigation of traditional medicinal plants commercialized in the markets of Mashhad, Iran. Avicenna J Phytomed.

[B8] Amiri MS, Joharchi MR, TaghavizadehYazdi ME (2014). Ethno-medicinal plants used to cure jaundice by traditional healers of Mashhad, Iran. Iranian J Pharm Res.

[B9] Azizi H, Keshavarzi M (2015). Ethnobotanical study of medicinal plants of Sardasht, Western Azerbaijan, Iran. J Herbal Drugs.

[B10] Bahmani M, Rafieian-Kopaei M, Avijgan M, Hosseini S, Golshahi H, Eftekhari Z, Gholizadeh GH (2012). Ethnobotanical studies of medicinal plants used by Kurdish owner's in south range of Ilam province, west of Iran. Am-Euras J Agric Environ Sci.

[B11] Barani H, Rahimpour S (2014). The dyeing procedures evaluation of wool fibers with prangos ferulacea and fastness characteristics. Advances in Materials Science and Engineering.

[B12] Delnavazi M R, Hadjiakhoondi A, Delazar A, Ajani Y, Tavakoli S, Yassa N (2015). Phytochemical and Antioxidant Investigation of the Aerial Parts of Dorema glabrum Fisch & CA Mey. Iranian J Pharm Res.

[B13] Dolatkhahi M, Ghorbani Nohooji M, Mehrafarin A, Amini Nejad G R, Dolatkhahi A (2012). Ethnobotanical study of medicinal plants in Kazeroon, Iran: Identification, distribution and traditional usage. J Med Plants.

[B14] Dolatkhahi M, Nabipour I (2014). Ethnobotanical Study of Medicinal Plants Used in the Northeast Latrine Zone of Persian Gulf. J Med Plants.

[B15] Emami SA, Nadjafi F, Amine GH, Amiri MS, Khosravi Mt, Nasseri M (2012). Les espèces de plantes médicinales utilisées par les guérisseurs traditionnels dans la province de Khorasan, nord-est de l'Iran. J Ethnopharmacol.

[B16] Emami SA, Aghazari F (2011). Iranian endemic phanerogams.

[B17] Ghorbani A (2005). Studies on pharmaceutical ethnobotany in the region of Turkmen Sahra, north of Iran (Part 1): General results. J Ethnopharmacol.

[B18] Ghorbani A, Naghibi F, Mosaddegh M (2006). Ethnobotany, ethnopharmacology and drug discovery. Iranian J Pharm Sci.

[B19] Ghorbani A, MirzaeiA-ghjeh-Qeshlagh F, Valizadeh-Yonjalli R (2014). Folk Herbal Veterinary Medicines of Zilberchay Watershed of East Azerbaijan (Iran). J Herbal Drugs.

[B20] Hooper D, Field H (1937). Useful plants and drugs of Iran and Iraq Field Museum of Natural History. Botanical Series.

[B21] Joharchi MR, Amiri MS (2012). Taxonomic evaluation of misidentification of crude herbal drugs marketed in Iran. Avicenna J Phytomed.

[B22] Khodayari H, Amani S, Amiri H (2015). Ethnobotanical study of medicinal plants in different regions of Khuzestan province.

[B23] Koohpayeh A, Ghasemi Pirbalouti A, Yazdanpanah Ravari MM, Pourmohseni Nasab E, Arjomand D (2011). Study the ethno-veterinary of medicinal plants in Kerman province, Iran. J Herbal Drugs.

[B24] Mardaninejad S, Janghorban M, Vazirpour M (2013). Collection and identification of medicinal plants used by the indigenous people of Mobarakeh (Isfahan), southwestern Iran. J Herbal Drugs.

[B25] Meeker JE, Elias JE, Heim JA (1993). Plants used by the Great Lakes Ojibwa Odanah.

[B26] Mirdeilami SZ, Barani H, Mazandarani M, Heshmati GA (2011). Ethnopharmacological survey of medicinal plants in Maraveh Tappe region, north of Iran. Iran J Plant Physiol.

[B27] Mosaddegh M, Naghibi F, Moazzeni H, Pirani A, Esmaeili S (2012). Ethnobotanical survey of herbal remedies traditionally used in Kohghiluyeh va Boyer Ahmad province of Iran. J ethnopharmacol.

[B28] Mozaffarian V, Assadi M, Khatamsaz M, Maasoumi AA (2007). Umbelliferae. Flora of Iran, No. 54..

[B29] Mozaffarian V (2007). A Dictionary of Iranian Plant Names.

[B30] Mozaffarian V (2013). Identification of medicinal and aromatic plants of Iran.

[B31] Naghibi F, Mosaddegh M, Mohammadi Motamed S, Ghorbani A (2005). Labiatae family in folk medicine in Iran: From ethnobotany to pharmacology. Iranian J Pharm Res.

[B32] Nazemiyeh H, Razavi SM, Delazar A, Asnaashari S, Khoi NS, Daniali S, Nahar L, Sarker SD (2009). Distribution Profile of Volatile Constituents in Different Parts of Astrodaucus orientalis (L) Drude. Rec Nat Prod.

[B33] Pae HO, Oh H, Yun YG, Oh GS, Jang SI, Hwang KM (2002). Imperatorin, a furanocoumarin from Angelica dahurica (Umbelliferae), induces cytochrome c-dependent apoptosis in human promyelocytic leukaemia, HL-60 cells. Pharmacol Toxicol.

[B34] Pimenov MG, Leonov MV (1993). The genera of the Umbelliferae.

[B35] Pirbalouti AG, Malekpoor F, Enteshari S, Yousefi M, Momtaz H, Hamedi B (2010). Antibacterial activity of some folklore medicinal plants used by Bakhtiari tribal in Southwest Iran. Int J Biol.

[B36] Ghasemi PA, Momeni M, Bahmani M (2013). Ethnobotanical study of medicinal plants used by Kurd tribe in Dehloran and Abdanan districts, Ilam province, Iran. Afr J Tradit Complement Altern Med.

[B37] Rajaei P, Mohamadi N, Motamed M (2012). Ethnobotanical Study of Medicinal Plants of Hezar Mountain Allocated in South East of Iran. Iranian J Pharm Res.

[B38] Sadeghi Z, Kuhestani K, Abdollahi V, Mahmood A (2014). Ethnopharmacological studies of indigenous medicinal plants of Saravan region, Baluchistan, Iran. J ethnopharmacol.

[B39] Sadeghi Z, Mahmood A (2014). Ethno-gynecological knowledge of medicinal plants used by Baluch tribes, southeast of Baluchistan, Iran. Rev Bras Farmacog.

[B40] Safa O, Soltanipoor MA, Rastegar S, Kazemi M, Dehkordi Kh, Ghannadi A (2013). An ethnobotanical survey on hormozgan province, Iran. Avicenna J Phytomed.

[B41] Safarnejad A, Abbasi M, Tabatabaei SM (2011). Agronomical and Botanical Characteristics of Cuminum setifolium (Boiss) Kos-Pol a Plant with Potentially Medicinal Applications. Notulae Sci Biol.

[B42] Sahranavard S, Ghafari S, Mosaddegh M (2014). Medicinal plants used in Iranian traditional medicine to treat epilepsy. Seizure.

[B43] Sajjadi S, Batooli H, Ghanbari A (2011). collection, evaluation and ethnobotany of Kashan medicinal plants.

[B44] Shafie-zadeh F (2002). Medicinal plants of Lorestan.

[B45] Sharififar F, Koohpayeh A, Motaghi MM, Amirkhosravi A, Puormohseni Nasab E, Khodashenas M (2010). Study the ethnobotany of medicinal plants in Sirjan, Kerman province, Iran. J Herbal Drugs.

[B46] Sharififar F, Moharam-Khani M, Moattar F, Babakhanloo P, Khodami M (2014). Ethnobotanical Study of Medicinal Plants of Joopar Mountains of Kerman Province, Iran. J Kerman Uni Med Sci.

[B47] Singh V, Jain DK (2007). Taxonomy of angiosperms.

[B48] Sonboli A, Eftekhar F, Yousefzadi M, Kanani MR (2005). Antibacterial activity and chemical composition of the essential oil of Grammosciadium platycarpum Boiss from Iran. Z Naturforsch [C].

[B49] Tahvilian R, Shahriari S, Faramarzi A, Komasi A (2014). Ethno-pharmaceutical Formulations in Kurdish Ethno-medicine. Iranian J Pharm Res.

[B50] Yazdanshenas H, Shafeian E, Nasiri M, Mousavi SA (2015). Indigenous knowledge on use values of Karvan district plants, Iran. Environ Dev Sustain.

[B51] Zargari A (1996). Medicinal Plants.

[B52] Zarshenas MM, Arabzadeh A, Tafti MA, Kordafshari G, Zargaran A, Mohagheghzadeh A (2013). Application of herbal exudates in traditional Persian medicine. Galen Med J.

